# Sevoflurane preconditioning induces tolerance to brain ischemia partially via inhibiting thioredoxin-1 nitration

**DOI:** 10.1186/s12871-018-0636-z

**Published:** 2018-11-17

**Authors:** Shiquan Wang, Yuheng Li, Jinlong Wei, Pei Li, Qianzi Yang

**Affiliations:** 1 Department of Anesthesiology and Perioperative Medicine, Xijing Hospital, Fourth Military Medical University, Xi’an, 710032 People’s Republic of China; 2 Department of Basic Medicine, Fourth Military Medical University, Xi’an, 710032 People’s Republic of China

**Keywords:** Sevoflurane, Preconditioning, Brain, Ischemia/reperfusion, Trx-1, Nitration

## Abstract

**Background:**

Sevoflurane preconditioning induces brain ischemic tolerance, but the mechanism remains poorly elucidated. Nitration is an important form of post-translational modification in pathological signaling. This study was to investigate the role of thioredoxin-1 (Trx-1) nitration in neuroprotection effect induced by sevoflurane preconditioning in a transient stroke model in rats.

**Methods:**

Adult male Sprague–Dawley rats were preconditioned with 2% sevoflurane or vehicle oxygen exposure, 1 h per day, for 5 consecutive days. At 24 h after the last exposure, rats were subjected to focal brain ischemia induced by middle cerebral artery occlusion (MCAO) for 90 min, followed by 72-h reperfusion. Trx-1 expression and activity, as well as the content of nitrotyrosine at penumbra were detected at 24 h after preconditioning and 2, 8, 24, 72 h after MCAO. Nitrated Trx-1 was examined by immunoprecipitation at 8 h after MCAO. The role of Trx-1 nitration in ischemic tolerance was assessed by administration of nitrated human-Trx-1 prior to MCAO. Neurological scores, brain infarct volumes and TUNEL staining were evaluated at 24 h after reperfusion.

**Results:**

Ischemic stroke decreased Trx-1 activity but not the expression in penumbra tissue. The content of nitrotyrosine was elevated after MCAO. Preconditioning with sevoflurane increased Trx-1 activity and reduced its nitration at 8 h after MCAO in comparison with vehicle preconditioning. The decrement of Trx-1 activity was correlated with its nitration level. Exogenous administration of nitrated human-Trx-1 reversed the brain ischemic tolerance of sevoflurane preconditioning, exacerbating brain infarct volume, neurobehavioral defects and apoptosis, while administration of human-Trx-1 had no effect on the sevoflurane preconditioning-induced neuroprotection.

**Conclusion:**

Ischemic stroke reduces Trx-1 activity via post-translational nitrative modulation in rats. Sevoflurane preconditioning induces brain ischemic tolerance and anti-apoptosis by partially preserving Trx-1 activity via inhibiting nitration.

## Background

Preconditioning with volatile anesthetics induces tolerance to cerebral ischemia/reperfusion injury in animals, and prevents neurologic complications such as perioperative stroke in patients [1]. Sevoflurane, one of the popular inhalational anesthetics, has shown the neuroprotective property in perioperative period [2, 3]. However, the mechanism of sevoflurane preconditioning is not fully understood.

The anti-oxidation and anti-apoptosis are the most important underlying pathways associated with the preconditioning-elicited neuroprotection, in which the activation of many signaling molecules and enzymes are altered directly or indirectly by the preconditioning interventions [3, 4]. The alteration occurs at gene level (transcriptional regulation) and/or the protein level (posttranslational modification). Extensive evidences have indicated that protein nitration is an important form of post-translational modification in pathological signaling since multiple intracellular proteins involved in oxidative stress and apoptosis are nitrated [5]. Interventions that modulate post-translational modification of protein have been proposed in the treatment of ischemia [5].

Thioredoxin − 1(Trx-1), a 12-KDa protein, ubiquitously expresses in all living cells, plays a pivotal role in cellular responses to cell growth, cell cycle, gene expression and apoptosis [6]. Trx-1 also acts as a powerful antioxidant in scavenging the reactive oxygen species (ROS), and is closely related to the pathogenesis of various oxidative stresses. Intraperitoneal administration of recombinant human Trx-1 (hTrx-1) protected brain damage from ischemic stroke [7], while reducing the level of Trx-1 by small interfering RNA deteriorated the cerebral injury [8]. In addition, the anti-apoptosis effect of Trx-1could be realized by interacting with apoptosis-regulating kinase-1 signaling [9, 10] .The recent studies have also authenticated that nitrative modification inactivated Trx-1 after heart ischemia, which resulted in myocardial apoptosis [11].

In present study, we investigated whether Trx-1 was involved in the neuroprotection induced by sevoflurane preconditioning in transient middle cerebral artery occlusion (MCAO) model in rats. We hypothesized that the nitration of Trx-1 after stroke could contribute to its loss of activity, and might be the mechanism underlying sevoflurane preconditioning-induced neuroprotection.

## Methods

All procedures involving the use of animals were approved by the ethics committee of animal experiment of the Fourth Military Medical University (Xi′an, China), and proceeded in accordance with the guidelines for animal experimentation of the same university. The Sprague Dawley rats were bought from Fourth Military Medical University and acclimatized for one week in a 12 h:12 h of light:dark cycle before interventions.

### Experimental design

Experiment 1. To test the time course of sevoflourane preconditioning on the alteration of Trx-1 after MCAO, rats were randomly divided into two groups (*n* = 15 for each group, 3 for each time point): Sevoflurane MCAO group (Sev MCAO) and Oxygen MCAO group (Oxy MCAO). Rats were subjected to MCAO at 24 h after sevoflurane or its vehicle oxygen preconditioning. Animals were decapitated to extract proteins at 24 h after last exposure of preconditioning without surgery as well as at 2 h, 8 h, 24 h and 72 h after MCAO, respectively. The expression of Trx-1 in brain ischemic penumbra tissue was examined by western blotting. The activity of Trx-1 at penumbra was measured by fluorescent plate with the spectrometer. The nitrotyrosine of protein in brain tissue was determined by chemilumineseence method.

Experiment 2. To assess the effects of sevoflurane preconditioning and MCAO ischemia/reperfusion on the nitration of Trx-1, animals were randomized into four groups (*n* = 4 for each group): Oxygen group (Oxy), Sevoflurane group (Sev), Oxygen MCAO group (Oxy MCAO) and Sevflurane MCAO group (Sev MCAO). Rats in Oxy and Sev groups underwent the sham operations, which didn’t induce ischemia. Meanwhile, rats in Oxy MCAO group and Sev MCAO group were subjected to MCAO at 24 h after preconditioning. The levels of nitrated Trx-1 were tested at 8 h after MCAO by immunoprecipitation, and the correlation between Trx-1 nitration and its activity was analyzed.

Experiment 3. To elucidate whether the enhancement of Trx-1 nitration could reverse the brain ischemic tolerance induced by sevoflurane, rats were randomly divided into four groups (*n* = 10 for each group, 7 for neurological scores and infarct volume, 3 for TUNEL staining): Oxy MCAO group, Sev MCAO group, Nitrated human-Trx-1 (3 N-Trx-1) group and human-Trx-1 (hTrx-1) group. Animals in Oxy and Sev MCAO groups were treated in the same way as those in Experiment 2. In 3 N-Trx-1 and hTrx-1 groups, sevoflurane-preconditioned rats received the nitrated hTrx-1 or hTrx-1 injection through tail vein at dose of 0.2 mg/kg, respectively, prior to MCAO. Neurological scores, brain infarct volumes and TUNEL staining were evaluated at 24 h after reperfusion.

### Sevoflurane preconditioning

The protocols of sevoflurane preconditioning were based on our previous publications [2]. In brief, rats were placed in a temperature-controlled, transparent and air-tight (30 × 30 × 20 cm^3^) chamber with a gas inlet port and an outlet port. During sevoflurane preconditioning, the box was flushed with 2% sevoflurane in 100% oxygen; As for oxygen preconditioning, the box was flushed with only 100% oxygen. The expired fraction of sevoflurane, oxygen, and carbon dioxide were monitored continuously (MP-60, Phillips Medical Systems, Best, The Netherlands). Soda-lime (Molecular products limited, Essex, United Kingdom) was placed at the bottom of container for carbon dioxide absorption. The sevoflurane preconditioning was performed for 1 h per day, repeated for five consecutive days.

### Focal cerebral ischemia and reperfusion

The animals had free access to water and food before the surgery. MCAO was operated as described previously [2]. SD rats were anesthetized with 60 mg/kg pentobarbital sodium (intraperitoneal injection), breathing spontaneously. Once the right common carotid artery and its branches were separated, a nylon monofilament (RWD, China) was inserted from the external carotid artery to internal carotid artery then to the right middle cerebral artery. Ninety minutes after occlusion, the filament was withdrawn to induce the reperfusion in the right middle cerebral artery territory. During the surgery, cerebral blood flow was monitored by laser-Doppler flowmetry (periflux 5000, perimedAB, Sweden). A reduction in cerebral blood flow of more than 80% baseline (pre-ischemia) was confirmed as the effective occlusion; otherwise, animals were excluded from the further experiments. Throughout the surgery, the temporal temperature was maintained at 37 °C ± 0.5 °C by a thermostatic blanket and a heating lamp. All the animals had a comparable surgical duration of time.

### Western blotting

Brain ischemic penumbra was dissected, and then homogenized in the RIPA lysis buffer (Beyotime, Nantong, China) containing whole proteinase inhibitor cocktail. A BCA protein assay kit (Beyotime, Nantong, China) was used to determine the protein concentration. Equivalent amount of protein (30 μg per lane) was loaded and separated by a 12% SDS-PAGE gel. After electrophoresis, the protein was transferred to a polyvinylidene difuoride (PVDF) membrane, which was then blocked with 2% bovine serum albumin (BSA) in TBST. The sample membrane was incubated overnight at 4 °C with the anti-Trx-1 (1:1000, Abcam, Cambridge, UK) and β-actin primary antibodies. At last, the membrane was incubated with horseradish peroxidase-conjugated secondary goat anti-rabbit antibody (1:5000, Pierce, Rockford, IL) for 1 h at room temperature. Analysis software program *Image J* was used to quantify the optical density of each band.

### Immunoprecipitation

The tissue was homogenized in the RIPA lysis buffer (Beyotime, Nantong, China) on ice. After centrifugation at 13000 rpm for 10 min, the supernatant was collected and protein concentration was measured by BCA protein assay kit (Beyotime, Nantong, China). Equivalent amount of protein was incubated with 2μg of mouse monoclonal anti-3-nitrotyrosine (Abcam, ab61392, UK) for 12 h with gentle rotation at 4 °C, and then added 50 μl of protein G/A beads for 3 h at 4 °C. Beads were then washed with cell lysis buffer for three times and the bound proteins were eluted with 2 × loading sample buffer and subjected to SDS-PAGE in 12% polyacrylamide gels, followed by blotting onto nitrocellulose filter membrane with the anti-Trx-1 antibody (Abcam, ab26320, UK). Immunoreactive bands were detected using Chemidoc MP imaging system (Bio-rad). Analysis software program *Image J* was used to quantify the optical density of each band.

### Nitrotyroysine assay

A nitrotyroysine assay kit (Millipore, lot 2,113,321, USA) was used to test the nitrotyrosine of the proteins. The procedure was performed in strict accordance with the instructions. In brief, the sample was homogenized in the RIPA lysis buffer (Beyotime, Nantong, China) on ice, and 50 μl of test sample or standard was incubated with 50 μl of 2 × anti-nitrotyrosine in the plate at 37 °C for 60 min. After washing, the plate was then incubated with 1 × anti-Rabbit lgG, HRP-conjugate (100 μl per well) at 37 °C for 60 min. Lately, the freshly prepared chemiluminescent substrate was added into each well and incubated at room temperature for 10 min. Chemidoc MP imaging system was used to quantify the optical density of the each well.

### The activity of Trx-1

Assay kits (IMCO, USA) were used to measure the activity of Trx-1. Specifically, Human-Trx-1 at different concentrations used to draw the standard curve by reacting with 10 μl TrxR and followed by incubation with 5 μl β-NADPH for 30 min at 37 °C in an incubator plate. Then 20 μl of the fluorescent substrate was added into each well for incubation for another 60 min. The emission at 545 nm after 520 nm excitation was recorded in a fluorescent plate reader at room temperature. The activity of samples was calculated by the fluorescence concentration and the standard curve.

### In vitro nitration of Trx-1

The hTrx-1 (Sigma, USA) was subjected to in vitro nitration with a modified procedure as described previously [11]. The purified hTrx-1 was dissolved in 0.1 μm phosphate buffer (pH = 7.4), of the final concentration at 50 μm. HTrx-1 was then incubated with Sin-1 (final concentration of 100 μM, Cayman Chemical, USA) at 37 °C for 30 min. The unreacted Sin-1 was removed by ultrafiltration through a 10-kDa cut-off membrane.

### Neurobehavioral evaluation and infarct assessment

According to the method of Garcia et al [12], neurological behaviors were assessed at 24 h after MCAO by an observer who was blinded to the animal grouping. Infarct volumes were measured immediately after Neurobehavioral evaluation. Rat brains were sliced into 2-mm coronal sections and stained by 2,3,5-triphenyltetrazolium chloride (1%; TTC, Sigma, USA).

### TUNEL staining

The cellular apoptosis was evaluated at 24 h post reperfusion by an in situ terminal deoxynucleotidyl-transferase-mediated 2′-deoxyuridine 5′-triphosphate nick-end labeling assay (TUNEL, Roche Diagnostics, Germany). Rat brains were fixed with paraformaldehyde and frozen with embedding agent, and then cut into 14 μm coronal sections from approximately 2 mm rostral to Bregma point. The slices were stained as the manufacture’s instruction. The positive cells were acquired by using a 40× objective lens in the ischemic penumbra, and the counting of positive cells was expressed as number per 100μm^2^. The ischemic penumbra area was defined according to the previous publications [3].

### Statistical analysis

**Fig. 1:**
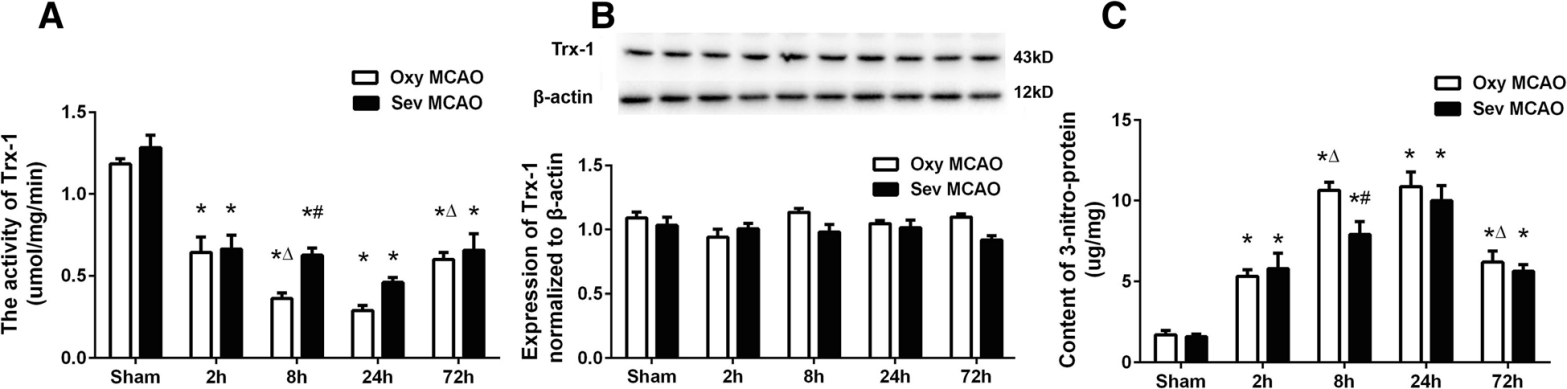
Alterations in activity and expression of Trx-1 and content of nitrotyrosine after sevoflurane preconditioning and MCAO. Ischemic penumbra areas of MCAO rats and corresponding tissues of non-ischemic rats were measured for the activity (**a**) and expression of Trx-1 (**b**) and level of nitrotyrosine (**c**) at different time points (*n* = 3 for each time point). ^*^P<0.05 vs. Sham with same Preconditioning,^△^*P*<0.05 vs. Previous time Point with same Preconditioning, ^#^*P*<0.05 vs. Oxy MCAO at the same time point

The software SPSS 12.0 for Windows (SPSS Inc., Chicago, IL) was used to analyze the data. All values were presented as mean ± SD, except for neurologic scores as median [interquartile range]. Values in Fig. 1 were analyzed by Two-way analysis of variance (ANOVA), and multiple comparisons within groups were performed by Bonferroni’s test. Values in other figures were analyzed by One-way ANOVA, and between-group differences were detected with Tukey post hoc test. The neurologic scores were analyzed by Kruskal-Wallis test followed by Mann-Whitney U test. Two-tailed values of *P* < 0.05 were considered statistically significant.

## Results

Since we used exactly the same the protocol for preconditioning as our previous studies [2], the physiologic variables at the end of the last preconditioning exposure and during surgery had been reported. Briefly, no difference in pH value, temporal temperature (T°C), partial pressure of oxygen (PaO_2_) and partial pressure of carbon dioxide (PaCO_2_) was observed between sevoflurane preconditioning and its vehicle oxygen groups.

### Sevoflurane preconditioning increased the activity but not the expression of Trx-1

To understand the role of Trx-1 in the sevoflurane preconditioning-induced neuroprotection, we observed the activity changes of Trx-1 after brain ischemic stroke. As shown in Fig. 1a, Trx-1 activity in Oxy MCAO group dramatically decreased at 2 h after MCAO (0.64 ± 0.13 vs. 1.18 ± 0.04, Oxy MCAO 2 h vs. Sham, *P* < 0.05), became even lower at 8 h (0.36 ± 0.04 vs. 0.64 ± 0.13, Oxy MCAO 8 h vs. Oxy MCAO 2 h, *P* < 0.05), maintained at a low level until 24 h, and then rose up to 72 h after brain ischemia. Preconditioned with sevoflurane, the activity of Trx-1 also reduced sharply at 2 h after MCAO (0.66 ± 0.12vs. 1.28 ± 0.11, Sev MCAO 2 h vs. Sham, *P* < 0.05), but the further significant decrease was not noted within 72 h after MCAO. Moreover, there was a significant increase of Trx-1 activity in sevoflurane-preconditioned rats compared with those in vehicle group at 8 h after ischemia insult (0.63 ± 0.06 vs. 0.36 ± 0.04, Sev MCAO 8 h vs. Oxy MCAO 8 h, *P* < 0.05).

We then examined the expression changes of Trx-1 protein by using western blotting technology. Surprisingly, the expression of Trx-1 didn’t alter at any time point during 72 h after MCAO, and no difference between Oxy MCAO and Sev MCAO groups was observed (Fig. 1b).

### The protein nitration was elevated by brain ischemia but reduced by sevoflurane preconditioning

To verify the hypothesis that the activity of Trx-1 could be regulated by post-translational nitration, we firstly compared the overall nitrotyrosine level between Oxy MCAO group and Sev MCAO group. The general nitrotyrosine concentration was markedly elevated by MCAO at 2 h (5.31 ± 0.56 vs. 1.71 ± 0.37, Oxy MCAO 2 h vs. Sham, *P* < 0.05) and peaked at 8 h (10.64 ± 0.69 vs. 5.31 ± 0.56, Oxy MCAO 8 h vs. Oxy MCAO 2 h, *P* < 0.05), then dropped at 72 h (6.19 ± 0.97 vs. 10.87 ± 1.26, Oxy MCAO 72 h vs. Oxy MCAO 24 h, *P* < 0.05). Sevoflurane preconditioning significantly decreased the content of nitrative proteins at 8 h after MCAO (7.90 ± 1.13 vs. 6.19 ± 0.97, Sev MCAO 8 h vs. Oxy MCAO 8 h, *P* < 0.05).

### Sevoflurane preconditioning inhibited the nitration of Trx-1 after MCAO

**Fig. 2:**
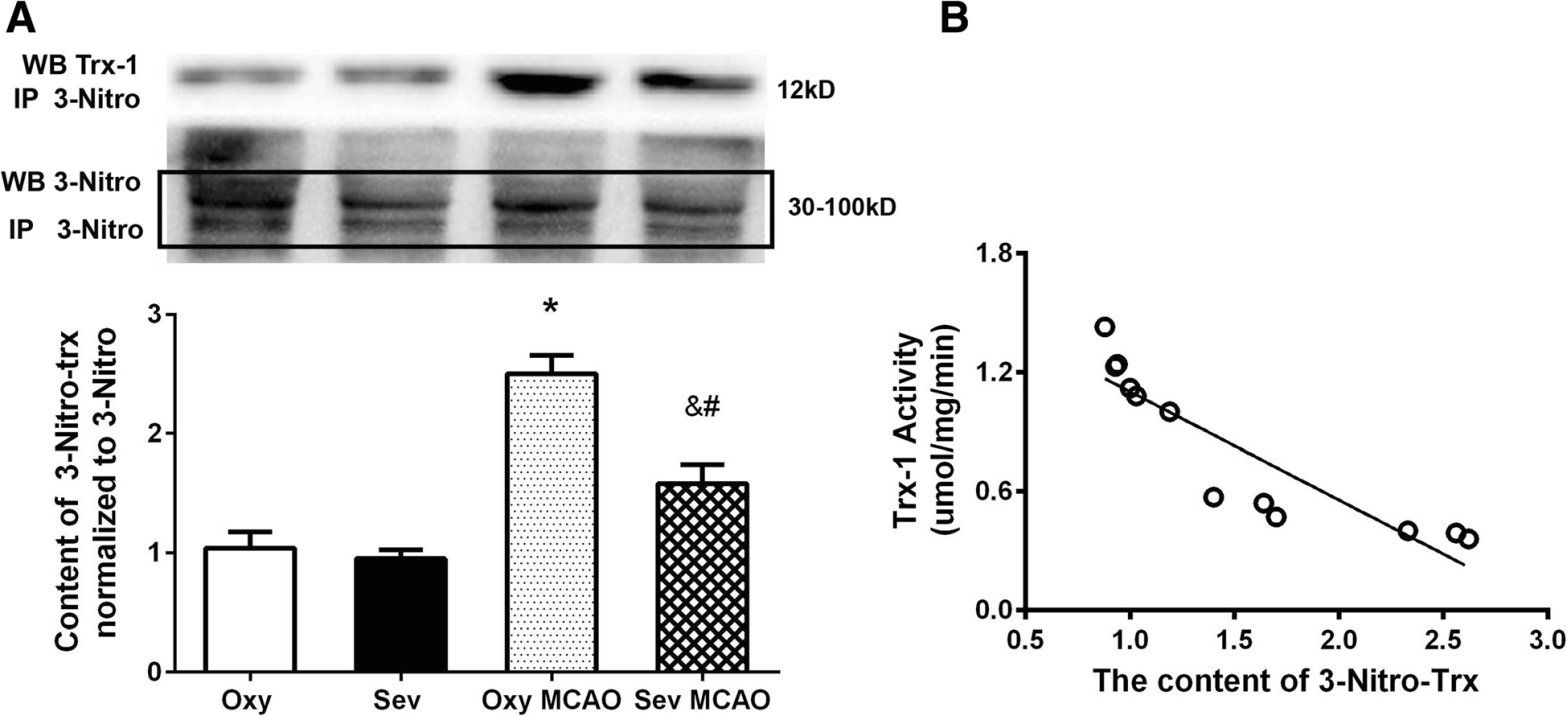
Nitration of Trx-1 in ischemic penumbra was reduced by sevoflurane preconditioning, negatively correlated with the activity of Trx-1. **a**. Sevoflurane preconditioning decreased the elevated expression of 3-nitro-Trx-1 at 8 h after MCAO. **b**. The content of 3-nitro-Trx-1 negatively correlated with the activity of Trx-1. ^*^P < 0.05 vs. Oxy, ^&^*P*<0.05 vs. Sev, ^#^*P*<0.05 vs. Oxy MCAO. Correlation coefficient − 0.8989, 95% confidence interval − 0.9716--0.6711, *P* < 0.001

Since the difference of Trx-1 activity between Oxy MCAO group and Sev MCAO occurred at 8 h after MCAO (Fig. 1), this time point was chosen for the further tests. By using immunoprecipitation technique, we examined the content of nitrative Trx-1 (3-Nitro-Trx-1) among four groups (Fig. 2a). At the end of preconditioning, sevoflurane had no effect on the Trx-1 nitration, compared with its vehicle (0.97 ± 0.30 vs. 1.04 ± 0.11 Oxy vs. Sev, *P* > 0.05). After brain ischemia, 3-Nitro-Trx-1 dramatically increased in vehicle group (Oxy MCAO vs. Oxy, 2.51 ± 0.13 vs. 0.97 ± 0.30, *P* < 0.05), but significantly reduced in Sev MCAO group (2.51 ± 0.13 vs. 1.58 ± 0.13, Oxy MCAO vs. Sev MCAO, *P* < 0.05), indicating sevoflurane exerted a down-regulative effect on the nitration of Trx-1 after MCAO.

### Post-MCAO nitration of Trx-1 correlated with Trx-1 activity

In order to clarify the relationship between nitration of Trx-1 and its activity, we did a regression analysis for these two factors. As plotted in Fig. 2b, the higher level of nitrated Trx-1, the lower activity was noted. There was a negative correlation between nitration content and activity of Trx-1 (correlation coefficient − 0.8989, 95% confidence interval − 0.9716--0.6711, *P* < 0.0001).

### Nitrated hTrx-1 reversed neuroprotection induced by sevoflurane preconditioning

**Fig. 3:**
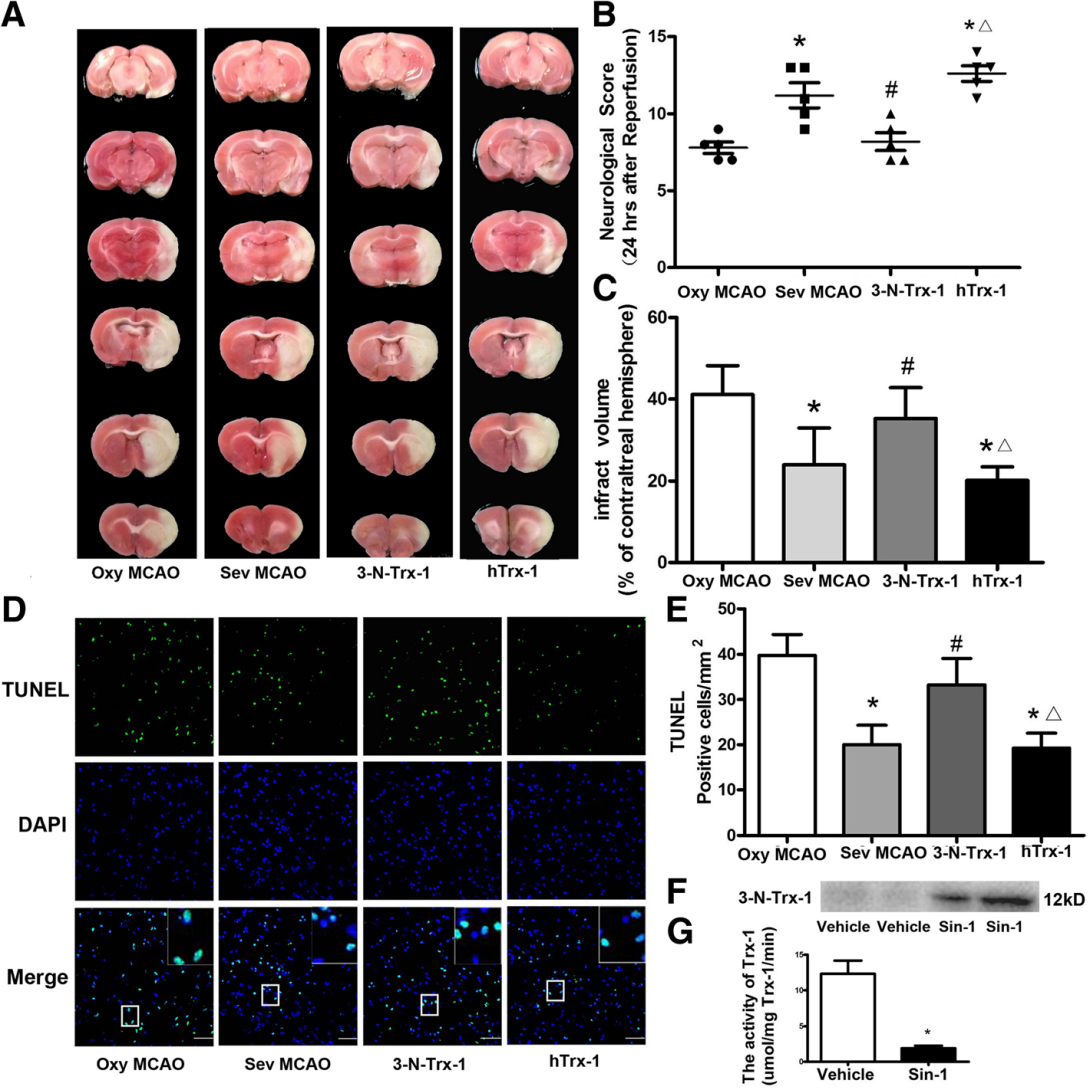
Nitrated hTRX-1 reversed neuroprotection induced by sevoflurane preconditioning. The administration of 3-N-Trx-1 enlarged brain infarct volume **a, c**, deteriorated the neurological results **b**, and aggravated apoptosis in Sev MCAO group **d, e**. Nitrated hTrx-1 was harvested in vitro by incubating Sin-1 with hTrx-1 and confirmed by western blotting after ultrafiltration **f**. Sin-1 almost completely inactivated the activity of Trx-1 **g**. ^*^P<0.05 vs. Oxy MCAO, ^#^*P* < 0.05 vs. Sev MCAO, ^△^*P* < 0.05 vs. 3-N-Trx-1. Bar = 50 μm

Sin-1, a peroxynitrite donor, has been proved to increase the nitration [11]. We incubated hTrx-1 with Sin-1 in vitro to harvest the nitrated hTrx-1 (3-N-Trx-1) by ultrafiltration. The expression of 3-N-Trx-1 was examined by western blotting (Fig. 3f), activity of Trx-1 in which was almost completely inhibited (Fig. 3g).

As illustrated in Fig. 3b, rats pre-treated with sevoflurane had better post-ischemic behavioral outcomes than those with oxygen vehicle (11 [10, 13] vs. 9 [8, 9], *P* < 0.05). However, this amelioration in neurological scoring was reversed by the 3-N-Trx-1 (8 [7, 8] vs. 11 [10, 13], *P* < 0.05), but not hTrx-1 (11 [10, 13] vs. 12 [10, 14], *P* > 0.05). 3-N-Trx-1 group and hTrx-1 group also showed significant difference in behavioral scores. Similarly, the brain infarct volume (Fig. 3a, c) in Sev MCAO group was significantly smaller than that in Oxy MCAO (23.96 ± 8.97 vs. 38.1 ± 9.61, *P* < 0.05), but 3-N-Trx-1 enlarged the infract size of brain (46.68 ± 5.57 vs. 23.96 ± 8.97, *P* < 0.05). The administration of hTrx-1 produced a smaller infract volume compared with 3-N-Trx-1 group (46.68 ± 5.57 vs. 20.16 ± 2.19, *P* < 0.05), but no difference was detected between Sev MCAO and hTrx-1.

The TUNEL positive cells were quantified at 24 h after reperfusion (Fig. 3d, e). Compared with Sev MCAO group, administration of 3-N-Trx-1 increased the number of TUNEL positive sells (20 ± 5 vs. 39 ± 4, *P* < 0.05), whereas hTrx-1 group had a similar amount of apoptosis (20 ± 5 vs. 19 ± 3, *P* > 0.05). The difference of apoptotic cell number between 3-N-Trx-1 and hTrx-1 groups were markedly shown in both fluorescent staining and statistics.

## Discussion

As a popular volatile anesthetic agent in clinic, sevoflurane has been reported as a potent neuroprotectant for perioperative stroke [2–4]. In current study, by using an in vivo model of transient focal cerebral ischemia in rats, we find that ischemic stroke decreased the activity but not the expression of Trx-1 in penumbra tissue. The decrement of activity was correlated with the nitration level of Trx-1. Preconditioning with sevoflurane mildly nitrified Trx-1 after brain ischemia/reperfusion and saved more activity of Trx-1. Exogenous administration of nitrated hTrx-1 reversed the brain ischemic tolerance of sevoflurane preconditioning, exacerbating brain infarction, neurobehavioral defects and neuronal apoptosis.

In mammalian cells, two isoforms of Trx have been identified: Trx-1 and Trx-2. Trx-1 is mostly cytosolic, while Trx-2 mainly expressed in mitochondria. Although the Trx system, including Trx, Trx reductase (TrxR), Trx peroxidase, and nicotinamide adenine dinucleotide phosphate, regulates cellular reduction/oxidation (redox) status and cell proliferation/cell survival [13], Trx-1 is most studied for its strong antioxidant function and stress-responsive character [14, 15].

The organ protective effect of Trx-1 against ischemia has been massively reported [6–8, 16, 17]. In both in vivo and in vitro animal models, Trx-1 has an explicit beneficial effect to heart, reducing infarct volume, improving ventricular function recovery, decreasing cellular apoptosis after ischemia [11, 16, 17]. In the brain, over-expression of Trx-1 and exogenous administration of hTrx-1 have been demonstrated to be protective against MCAO-induced cerebral ischemia damage [18–21]. Down-regulation of Trx-1 enhances the MCAO-produced brain damage and oxidation injury [8]. The underlying mechanism appears to be related to the inhibition of p38-kinase activation or phosphoinositide 3-kinase (PI3K) pathway [22, 23]. However, little evidence has been shown for the dynamic changes of Trx-1 in an ischemia brain.

In this study, we tested the alteration of Trx-1 in ischemic penumbra tissues at multiple time points within 72 h after brain ischemia, surprisingly found the expression of Trx-1 protein didn’t change before or after transient MCAO. As far as we noticed, this could be the first report on negative changes of Trx-1 expression after brain ischemia. Takagi and colleagues examined the changes of Trx in both a permanent and a transient focal cerebral ischemia model induced by MCAO [20, 24], and they found both Trx immunoreactivity and mRNA increased at 24 h after ischemia. Furthermore, another research also demonstrated the Trx-1 expression in ischemic cortex was elevated after 60 min MCAO [8]. However, these evidences in brain seem not to be in parallel with what has been discovered in hearts, since Trx-1 expression was reported to be reduced in myocardial tissue after 30 min ischemia followed by 2 h reperfusion [25]. The difference between our experiment and others could be attributed to the MCAO duration, whether or not with preconditioning, animals’ weight and age, selected time points, location of samples as well as the definition of ischemic penumbra. Since these parameters are not comparable, we may not be able to draw a universal conclusion on the dynamic changes of Trx-1 expression after brain ischemia/reperfusion yet. Our results indicate that Trx-1 protein level in ischemic penumbra area doesn’t alter within 72 h after a 90 min-MCAO.

Besides expression, we also tested the activity of Trx-1 before and after MCAO, which was barely reported in previous studies. We found Trx-1 activity decreased from 2 h after MCAO and maintained at an even lower level from 8 h to 24 h, and then was elevated at 72 h after brain ischemia. This result indicates the activity of Trx-1 might be more sensitive than its expression to the brain ischemia insult, indicating the activity as a better index for the alteration of Trx-1 for further investigation.

Trx-1 was not only reduced after ischemia but also changed by sevoflurane intervention. As expected, we found that sevoflurane preconditioning delayed the latency that Trx-1 getting to the lowest activity after MCAO, and elevated the Trx-1 activity at 8 h after ischemia insult. This result coincides with the previous observations, in which the number of Trx-1 immunoreactive cells increased in mild hypobaric hypoxia preconditioned rats, but this elevation was only observed at 3 h after severe hypobaric hypoxia insult, whereas at 24 h, there was no remarkable difference in the Trx immunoreactivity between pre- and non-preconditioned animals [26, 27].

Although sevoflurane preconditioning preserved more activity of Trx-1 after MCAO, it didn’t induce any changes in Trx-1 activity at 24 h after the last preconditioning exposure prior to ischemia, in comparison with vehicle group. This phenomenon was also seen in Stroev’s study [26], where preconditioning with three sessions of moderate hypoxia significantly increases the expression of Trx-1 in the rat hippocampus by 3 h after subsequent acute severe hypoxia, but no change of Trx-1 could be detected in hippocampus at 3 h after preconditioning. Similarly, in our previous publication [3], we examined the expression of Notch signaling receptor after sevoflurane preconditioning without brain ischemia, and found it increased at 2 h and the elevation disappeared at 24 h afterwards. Thus, the alteration of Trx-1 after preconditioning could probably be dynamic, neither expression nor activity is associated with the accumulation of this protein after preconditioning, but with an induced modulation of the reaction to subsequent ischemia/reperfusion injury.

The effect of Trx-1 is not only determined by expression but also regulated by post-translational modification. A total of four forms of post-translational modifications in Trx have been identified, including oxidation, glutathionylation, S-nitrosylation, and nitration. The first three are related to cysteine residues, whereas nitration is particularly modified at the tyrosine residue. In mammals, nitration of Trx-1 occurs at Tyr^49^. Tao et al [11] revealed that nitration of Tyr^49^ is a novel post-translational modification that inhibits Trx-1 function by conformational change. They also demonstrated that ONOO^−^, but not NO or ROS, is responsible for nitrative inactivation of Trx-1.

In this study, we have demonstrated that Trx-1 is subject to nitrative modification after brain ischemia/reperfusion insult with following evidence: the nitration content of overall proteins is elevated; the decrement of activity of Trx-1 is correlated with post-translational nitration of Trx-1; sevoflurane preconditioning inhibits nitration of Trx-1 after brain ischemia. Previously, it was reported that suppression on ONOO^−^ formation by either hTrx-1 or an ONOO^−^ scavenger uric acid reduced cerebral infarct size in mice subjected to cerebral ischemia, and peroxynitrite donor Sin-1 markedly attenuated hTrx-1-induced antioxidative/antinitrative effect [19]. Although nitration often takes place along with oxidation, we directly administered nitrated hTrx-1 exogenously and found it abolished brain ischemic tolerance induced by sevoflurane preconditioning. These evidences strongly suggest the nitrative modification of Trx-1 could be a key mechanism of sevoflurane-reconditioning neuroprotection, as well as a pivotal regulative target for ischemia injury. Furthermore, nitrated hTrx-1 also increased the number of apoptotic cells in the sevoflurane preconditioned animals, indicating Trx-1 could be involved in the brain ischemic damage and tolerance via an anti-apoptosis pathway.

Interestingly, although hTrx-1 is well known for exerting a marked protection against stroke [18, 19], hTrx-1 in sevoflurane-preconditioned rats didn’t show any further effects in neuroprotection, even when there was a slight tendency to better outcomes of neurologic scores and infarct volume. It could be related to the less damage of blood-brain-barrier in sevoflurane-preconditioned rats, which give less chance for hTrx-1 to arrive at the ischemic area [18]. Another possible reason is the activation of Trx-1 in the brain may have a ceiling effect in neuroprotection, but surely this needs more data to confirm.

Some limitations in this study need to be noted. Because of the investigation focus was on the comparison between sevoflurane preconditioning and its vehicle, oxygen but not blank control was used. Pure oxygen preconditioning may also have an effect on Trx-1 activity compared with non-preconditioned rats. However, based on our previous studies, oxygen control has no neuroprotective effect on brain ischemia/reperfusion injury, and it doesn’t alter Mn-SOD or GSH-px activity at 24 h after preconditioning, which keep a cross-talk relationship with Trx-1 and also are modulated by nitration [28]. It could be reasonable to postulate that oxygen control would not cause the changes in Trx-1 accumulation after preconditioning, but for the further study, we might set a non-preconditioned control to get more solid evidence. Moreover, brain ischemia/reperfusion imposes multiple stresses; more than one post-translational modification could be responsible for Trx-1 activity. Although we revealed the down-regulative effect of sevoflurane preconditioning on nitration of Trx-1, other modifications could not be excluded.

## Conclusion

The current study demonstrated Trx-1activity was decreased by transient focal cerebral ischemia in MCAO model in rats via posttranslational nitrative modulation, and sevoflurane-preconditioning induced brain ischemic tolerance and anti-apoptosis by partially preserving Trx-1activity via inhibiting nitration. The posttranslational nitrative modulation of Trx-1 may represent a new and potential therapeutic target for neuroprotective mechanisms of sevoflurane preconditioning.

## Abbreviations

hTrx-1: Human-Trx-1
MCAO: Middle cerebral artery occlusion
Oxy MCAO: Oxygen MCAO
Oxy: Oxygen
Sev MCAO: Sevoflurane MCAO
Sev: Sevoflurane
Trx-1: Thioredoxin-1

## References

[1] Zwerus R, Absalom A (2015). Update on anesthetic neuroprotection. Curr Opin Anaesthesiol.

[2] Yang Q, Dong H, Deng J, Wang Q, Ye R, Li X, Hu S, Dong H, Xiong L (2011). Sevoflurane preconditioning induces neuroprotection through reactive oxygen species-mediated up-regulation of antioxidant enzymes in rats. Anesth Analg.

[3] Yang Q, Yan W, Li X, Hou L, Dong H, Wang Q, Dong H, Wang S, Zhang X, Xiong L (2012). Activation of canonical notch signaling pathway is involved in the ischemic tolerance induced by sevoflurane preconditioning in mice. ANESTHESIOLOGY.

[4] Cai M, Tong L, Dong B, Hou W, Shi L, Dong H (2017). Kelch-like ECH-associated protein 1-dependent nuclear factor-E2-related factor 2 activation in relation to Antioxidation induced by sevoflurane preconditioning. ANESTHESIOLOGY.

[5] Radi R (2018). Oxygen radicals, nitric oxide, and peroxynitrite: redox pathways in molecular medicine. Proc Natl Acad Sci U S A.

[6] D'Annunzio V, Perez V, Boveris A, Gelpi RJ, Poderoso JJ (2016). Role of thioredoxin-1 in ischemic preconditioning, postconditioning and aged ischemic hearts. Pharmacol Res.

[7] Wang B, Tian S, Wang J, Han F, Zhao L, Wang R, Ning W, Chen W, Qu Y (2015). Intraperitoneal administration of thioredoxin decreases brain damage from ischemic stroke. Brain Res.

[8] Li L, Zhu K, Liu Y, Wu X, Wu J, Zhao Y, Zhao J (2015). Targeting thioredoxin-1 with siRNA exacerbates oxidative stress injury after cerebral ischemia/reperfusion in rats. NEUROSCIENCE.

[9] Liu Y, Min W (2002). Thioredoxin promotes ASK1 ubiquitination and degradation to inhibit ASK1-mediated apoptosis in a redox activity-independent manner. Circ Res.

[10] Branco V, Coppo L, Sola S, Lu J, Rodrigues C, Holmgren A, Carvalho C (2017). Impaired cross-talk between the thioredoxin and glutathione systems is related to ASK-1 mediated apoptosis in neuronal cells exposed to mercury. Redox Biol.

[11] Tao L, Jiao X, Gao E, Lau WB, Yuan Y, Lopez B, Christopher T, RamachandraRao SP, Williams W, Southan G (2006). Nitrative inactivation of thioredoxin-1 and its role in postischemic myocardial apoptosis. CIRCULATION.

[12] Garcia JH, Wagner S, Liu KF, Hu XJ (1995). Neurological deficit and extent of neuronal necrosis attributable to middle cerebral artery occlusion in rats. Statistical validation STROKE.

[13] Maulik N, Das DK (2008). Emerging potential of thioredoxin and thioredoxin interacting proteins in various disease conditions. Biochim Biophys Acta.

[14] Yao P, Chen X, Yan Y, Liu F, Zhang Y, Guo X, Xu B (2014). Glutaredoxin 1, glutaredoxin 2, thioredoxin 1, and thioredoxin peroxidase 3 play important roles in antioxidant defense in Apis cerana cerana. Free Radic Biol Med.

[15] Lee BW, Jeon BS, Yoon BI (2018). Exogenous recombinant human thioredoxin-1 prevents acetaminophen-induced liver injury by scavenging oxidative stressors, restoring the thioredoxin-1 system and inhibiting receptor interacting protein-3 overexpression. J Appl Toxicol.

[16] Yin T, Hou R, Liu S, Lau WB, Wang H, Tao L (2010). Nitrative inactivation of thioredoxin-1 increases vulnerability of diabetic hearts to ischemia/reperfusion injury. J Mol Cell Cardiol.

[17] Jakobs P, Serbulea V, Leitinger N, Eckers A, Haendeler J (2017). Nuclear factor (erythroid-derived 2)-like 2 and Thioredoxin-1 in atherosclerosis and ischemia/reperfusion injury in the heart. Antioxid Redox Signal.

[18] Hattori I, Takagi Y, Nakamura H, Nozaki K, Bai J, Kondo N, Sugino T, Nishimura M, Hashimoto N, Yodoi J (2004). Intravenous administration of thioredoxin decreases brain damage following transient focal cerebral ischemia in mice. Antioxid Redox Signal.

[19] Ma YH, Su N, Chao XD, Zhang YQ, Zhang L, Han F, Luo P, Fei Z, Qu Y (2012). Thioredoxin-1 attenuates post-ischemic neuronal apoptosis via reducing oxidative/nitrative stress. Neurochem Int.

[20] Takagi Y, Horikawa F, Nozaki K, Sugino T, Hashimoto N, Yodoi J (1998). Expression and distribution of redox regulatory protein, thioredoxin during transient focal brain ischemia in the rat. Neurosci Lett.

[21] Zhou F, Gomi M, Fujimoto M, Hayase M, Marumo T, Masutani H, Yodoi J, Hashimoto N, Nozaki K, Takagi Y (2009). Attenuation of neuronal degeneration in thioredoxin-1 overexpressing mice after mild focal ischemia. Brain Res.

[22] Jin R, Gao Y, Zhang S, Teng F, Xu X, Aili A, Wang Y, Sun X, Pang X, Ge Q (2015). Trx1/TrxR1 system regulates post-selected DP thymocytes survival by modulating ASK1-JNK/p38 MAPK activities. Immunol Cell Biol.

[23] Wu X, Li L, Zhang L, Wu J, Zhou Y, Zhou Y, Zhao Y, Zhao J (2015). Inhibition of thioredoxin-1 with siRNA exacerbates apoptosis by activating the ASK1-JNK/p38 pathway in brain of a stroke model rats. Brain Res.

[24] Takagi Y, Tokime T, Nozaki K, Gon Y, Kikuchi H, Yodoi J (1998). Redox control of neuronal damage during brain ischemia after middle cerebral artery occlusion in the rat: immunohistochemical and hybridization studies of thioredoxin. J Cereb Blood Flow Metab.

[25] Turoczi T, Chang VW, Engelman RM, Maulik N, Ho YS, Das DK (2003). Thioredoxin redox signaling in the ischemic heart: an insight with transgenic mice overexpressing Trx1. J Mol Cell Cardiol.

[26] Stroev SA, Tyul'Kova EI, Glushchenko TS, Tugoi IA, Samoilov MO, Pelto-Huikko M (2009). Thioredoxin-1 expression levels in rat hippocampal neurons in moderate hypobaric hypoxia. Neurosci Behav Physiol.

[27] Stroev SA, Tjulkova EI, Gluschenko TS, Rybnikova EA, Samoilov MO, Pelto-Huikko M (2004). The augmentation of brain thioredoxin-1 expression after severe hypobaric hypoxia by the preconditioning in rats. Neurosci Lett.

[28] Guo W, Adachi T, Matsui R, Xu S, Jiang B, Zou MH, Kirber M, Lieberthal W, Cohen RA (2003). Quantitative assessment of tyrosine nitration of manganese superoxide dismutase in angiotensin II-infused rat kidney. Am J Physiol Heart Circ Physiol.

